# Confocal Laser Scanning Microscope Imaging of Custom-Made Multi-Cylinder Phantoms: Theory and Experiment

**DOI:** 10.3390/s23104945

**Published:** 2023-05-21

**Authors:** David Hevisov, Felix Glöckler, Felix Ott, Alwin Kienle

**Affiliations:** Institut für Lasertechnologien in der Medizin und Meßtechnik an der Universität Ulm, Helmholtzstr. 12, D-89081 Ulm, Germanyalwin.kienle@ilm-ulm.de (A.K.)

**Keywords:** confocal laser scanning microscopy, multi-scattering, cylindrical scatterer, Monte Carlo simulation, 3D direct laser writing

## Abstract

In this work, the image formation in a confocal laser scanning microscope (CLSM) is investigated for custom-made multi-cylinder phantoms. The cylinder structures were fabricated using 3D direct laser writing and consist of parallel cylinders with radii of 5 and 10 μm for the respective multi-cylinder phantom, with overall dimensions of about $200\times 200\times 200\;\mu m^{3}$. Measurements were performed for different refractive index differences and by varying other parameters of the measurement system, such as pinhole size or numerical aperture (NA). For theoretical comparison, the confocal setup was implemented in an in-house developed tetrahedron-based and GPU-accelerated Monte Carlo (MC) software. The simulation results for a cylindrical single scatterer were first compared with the analytical solution of Maxwell’s equations in two dimensions for prior validation. Subsequently, the more complex multi-cylinder structures were simulated using the MC software and compared with the experimental results. For the largest refractive index difference, i.e., air as the surrounding medium, the simulated and measured data show a high degree of agreement, with all the key features of the CLSM image being reproduced by the simulation. Even with a significant reduction in the refractive index difference by the use of immersion oil to values as low as 0.005, a good agreement between simulation and measurement was observed, particularly with respect to the increase in penetration depth.

## 1. Introduction

Confocal microscopy is a widely used imaging technique whose primary virtue lies in its ability to provide optical sectioning. This is achieved by focusing a point source of light onto a specific plane in the scattering medium. The reflected light is then focused onto a pinhole preventing light from out-of-focus areas, which has typically been scattered multiple times and thus is blurring the image, from reaching the detector. This allows for a significant increase in contrast compared to a conventional microscope [1]. The illuminated spot is then swept over the sample to produce a complete two-dimensional image, or even a three-dimensional image stack, by shifting the focal plane along the axial direction [2]. In the present work, a confocal laser scanning microscope (CLSM) is being considered, which, as the name suggests, uses a focused laser beam to scan the sample. The CLSM is a well-established imaging technique in various life sciences [3,4,5] but is also used in other fields, such as materials characterisation [6,7,8].

From a theoretical point of view, Monte Carlo (MC) methods have become the gold standard for describing light propagation in turbid media [9]. It is therefore not surprising to find that MC simulations are also applied in the study of confocal imaging [10,11,12,13,14]. In particular, Schmitt et al. [10] investigated the performance of a confocal microscope for probing a mirror embedded in scattering media. To this end, the light propagation has been modelled using an MC simulation where the beam path through the confocal optics was realised using geometric optics. By varying different setup parameters, such as the pinhole size or the axial mirror position, the simulated detector response was compared to the measured one. Chen et al. [11] used MC simulations to compare the imaging performance of different confocal microscope designs, also considering a mirror positioned in turbid media. Furthermore, Blanca et al. [12] implemented an MC-based numerical model to evaluate the image formation through scattering media for a confocal fluorescence microscope, specifically comparing two-photon and single-photon excitation. The investigation includes, inter alia, the influence of the object depth and the pinhole radius on the detected signal for an optical system that is focused on a perfectly reflecting sample. Mowla et al. [13] presented a technique that utilises confocal microscopy and laser Doppler flowmetry to combine the morphological and functional information provided by each method, to improve the imaging of malignant tumours. Therefore, MC simulations were used to image through a numerical multi-layer model of dermal tissue containing an encapsulated tumour. Finally, Carles et al. [14] proposed a hybrid method that incorporates the advantages of MC simulations and those of commercial ray-tracing software for optical systems. The MC method was used to model the light propagation in turbid media, whereas ray-tracing techniques were utilised to model complex optical systems. For example, on the basis of known 3D geometries, the imaging of retinal vasculature with a confocal scanning laser ophthalmoscope has been simulated.

However, Monte Carlo-based studies to date have mainly focused on theoretical aspects, with the direct comparison of full two-dimensional scans with experimental data being rather rare. In order to make such a comparison, a precise knowledge of the geometric and optical properties of the sample under investigation is of great advantage. Following this line of thought, image formation for different custom-made single scatterers has already been studied in a previous work [15]. By means of two-photon polymerisation, 3D direct laser writing (3D-DLW) enables the production of customised single scattering particles or even complex multi-scattering phantoms in a wide range of different geometries, while the dimensions of the 3D printed model can range from less than 100 nm up to 1 mm [16]. Thus, in our previous study, the 3D printed single scatterers were measured using a CLSM [15] and, in the case of the sphere, compared with theoretical calculations following an algorithm described in [17]. For the more complex single scatterers, i.e., platonic bodies, however, only a simple 2D-based Monte Carlo ray tracer was used to contribute to a better understanding of the individual image features.

In this work, a comparison between experiment and theory was now also carried out for complex multi-scattering phantoms. For this purpose, an in-house developed tetrahedron-based and GPU-accelerated Monte Carlo software [18] was extended by a CLSM implementation to describe the three-dimensional light propagation in the confocal setup for samples of arbitrary geometry. For comparison, multi-cylinder phantoms were fabricated using 3D-DLW with individual cylindrical scatterer radii of 5 and 10 μm and a total dimension of the phantoms of approximately $200\times 200\times 200\;\mu m^{3}$. In addition, the 2 phantom geometries, consisting of 5 and 10 μm radii cylinders, were each produced with 2 different photoresists to analyse a change in refractive index. To obtain further differences in refractive index, measurements were performed both in air and in immersion oil. To the best of our knowledge, this study is the first to compare CLSM experiments with simulations for a microscopic phantom with defined scatterer positions.

The paper is structured as follows. In Section 2, the experimental and theoretical methods relevant to this work are briefly introduced. More specifically, the setup of the CLSM, the fabrication of the multi-cylinder phantoms using 3D-DLW, the Monte Carlo implementation of the CLSM and the Maxwell theory used for validation are described. Then, in Section 3, the results of our work are presented and discussed in three parts. First, a theoretical comparison is made with the analytical solution of Maxwell’s equations to validate the Monte Carlo software. The comparison between the experiment and MC simulation for the fabricated multi-cylinder phantoms under variation of the refractive index difference follows. Lastly, the influence of the NA and pinhole size are experimentally evaluated. To conclude, the key findings of this paper are summarised in Section 4.

## 2. Materials and Methods

### 2.1. Confocal Laser Scanning Microscope

The measurements in this work were performed on the same CLSM (TCS SP8, Leica Microsystems GmbH, Wetzlar, Germany) and in the same manner as described by Lohner et al. [15]. The laser wavelength used was 552 nm and measurements were performed with several objectives. Apart from the previously described air objective with a NA of 0.6 (HCX PL FLUOTAR L 40×/0.60 CORR, Leica Microsystems GmbH), an oil objective with NA=1.4 (HC PL APO 63×/1.4 OIL CS2, Leica Microsystems GmbH) and other air objectives with NA=0.4 (LD Plan-NEOFLUAR 20×/0.4 Korr, Carl Zeiss AG, Jena, Germany) and NA=0.3 (HCX PL FLUOTAR 10×/0.3, Leica Microsystems GmbH) were used.

### 2.2. Custom-Made Multi-Cylinder Phantoms

**Figure 1:**
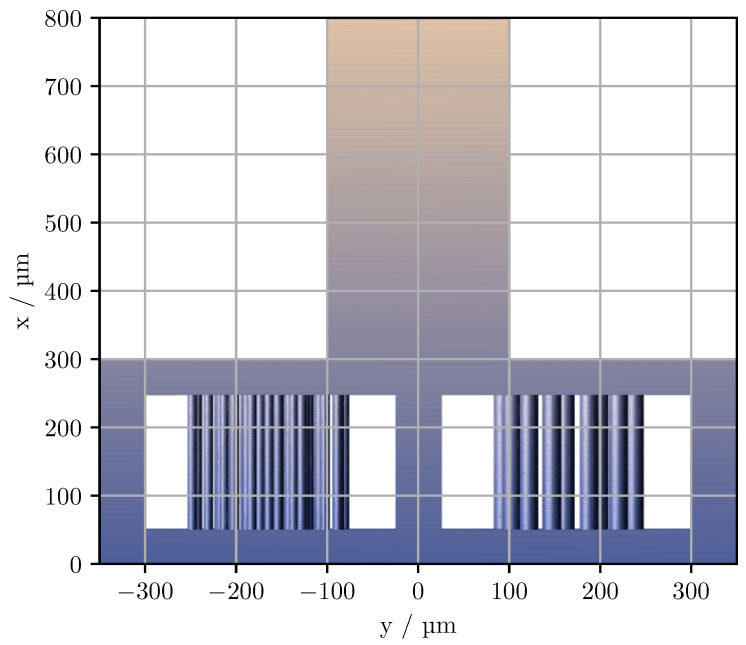
The cylinder phantom depicted with the preprint tool DeScribe (Nanoscribe GmbH). The glass substrate on which the structure is printed is at x=0.

Phantom models were created by randomly distributing cylinders within a $200\times 200\;\mu m^{2}$ slab in the *y*-*z* plane, as presented by Ott et al. [19]. The models were created for 2 different cylinder diameters, 10 μm and 20 μm, with the total amount of space occupied by the cylinders being approximate 10%. The cylinders were then printed vertically within a support frame to a height of x=200 μm using a commercial two-photon polymerisation printer (Photonic Professional GT+, Nanoscribe GmbH, Eggenstein-Leopoldshafen, Germany). The practical volume of the cylinder phantoms, excluding the support structures, is therefore around ${\left(200\;\mu m\right)}^{3}$. As shown in Figure 1, the frame was enlarged in the *y*-direction to avoid shadowing effects on the sides of the cylinder distribution. In the view shown in Figure 1, the optical axis of the incident light is along the *z*-axis during the acquisition of the CLSM images.

A handle was also printed on the top of the frame, which was used to turn the phantoms over and glue them to the substrate. The supporting structures, more specifically the frame and top handle, were printed using a shell and scaffold printing process and then flood-cured with UV light. The cylinders themselves were printed as full bodies using a single contour line. The slicing distance and the hatching distance were set to 0.25 μm, and a line speed of 10,000 μm $s^{-1}$ was used. Two phantoms were prepared in this way using different resins (IP-S and IP-n162, Nanoscribe GmbH, Germany). They have significantly different refractive indices at 552 nm, namely n=1.5133 for IP-S and n=1.6285 for IP-n162 [20]. The laser power was set to 25 mW for the IP-S printing process and 37.5 mW for the IP-n162.

### 2.3. Monte Carlo Implementation of the CLSM

MC simulations provide a numerical solution to the radiative transport equation (RTE). In this statistical method, a large number of independent energy packets, called photons in Monte Carlo jargon, are propagated along geometric rays through the medium under consideration. For the description of the light propagation, various interactions with the medium, such as scattering, absorption, reflection and refraction, can be taken into account. However, since effects such as diffraction due to the wave nature of light cannot be described, deviations from a real system are to be expected, especially when the dimensions of the object under consideration approach the scale of the wavelength.

**Figure 2:**
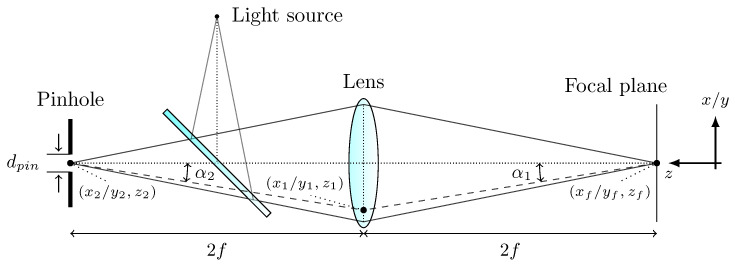
Geometry of the confocal configuration used in the MC simulation. The beam path is projected onto the *x*-*z* or *y*-*z* plane, assuming cylindrical symmetry along the *z*-axis. The dashed line illustrates the detection path through the optical system for an exemplary light ray.

For the comparison between theory and experiment, a numerical CLSM model was implemented in a proprietary tetrahedron-based and GPU-accelerated MC software [18]. By modelling the samples to be simulated as a tetrahedral mesh, geometries of almost any complexity can be considered. Furthermore, GPU parallelisation is of great benefit in a computationally intensive scenario such as this, where only a few reflected photons reach the pinhole. As previously mentioned, the CLSM produces a two-dimensional image by scanning the focus of light across the plane of interest. This means that for each pixel of the final simulated 2D scan, a separate MC simulation must be performed. This involves propagating the photons for the respective focal point ${\vec{r}}_{f}=\left(x_{f},y_{f},z_{f}\right)$ through the optical system and the object to be scanned. The geometry of the numerically implemented confocal system is shown schematically in Figure 2.

Before the simulation, the *x*- and *y*-interval and the number of pixels n×m to be sampled are defined. This results in the scan positions $y_{f}\in \left\{y_{1},\cdots ,y_{n}\right\}$ and $z_{f}\in \left\{z_{1},\cdots ,z_{m}\right\}$ for the *y* and *z* coordinates, respectively. In the following simulations, only the *y*-*z* plane is displayed due to the alignment of the cylinders parallel to the *x*-axis. Additionally, the desired size of the pinhole $d_{\mathrm{pin}}$ and the NA of the lens have to be specified as well. Given the focal length *f* of the lens, the lens diameter $d_{\mathrm{lens}}$ is then calculated using Equation (1) to match the given NA:

(1) $$d_{\mathrm{lens}}=\frac{\mathrm{NA}\;\cdot \;4f}{n_{s}\;\cdot \;\sqrt{1-{\left(\frac{\mathrm{NA}}{n_{s}}\right)}^{2}}}\;,$$

where $n_{s}$ is the refractive index of the surrounding medium.

At the start of the simulation, for a focal point ${\vec{r}}_{f}=\left(x_{f},y_{f},z_{f}\right)$, the lens is positioned according to the geometry shown in Figure 2. No beam splitter is required for the numerical implementation, so the photons are launched directly from the lens. The start positions $\left(x_{0},y_{0},z_{f}+2f\right)$ on the lens are determined based on two equally distributed random variables $\xi_{1},\xi_{2}\in \left[0,1\right]$ and are sampled evenly in the *x*-*y* plane bounded by the radius of the lens:

(2) $$r_{0}=r_{\mathrm{lens}}\;\cdot \;\sqrt{\xi_{1}}\;,$$

(3) $$\phi_{0}=2\pi \;\cdot \;\xi_{2}.$$

For a lens centred at $\left(x_{f},y_{f},z_{f}+2f\right)$, a random starting position ${\vec{r}}_{0}$ is thus given by

(4) $${\vec{r}}_{0}=\left(\begin{array}{c} x_{f}+r_{0}\;\cdot \;\cos\phi_{0}\\ y_{f}+r_{0}\;\cdot \;\sin\phi_{0}\\ z_{f}+2f \end{array}\right).$$

Assuming a geometric focus at exactly one point, the initial direction ${\vec{s}}_{0}$ of the corresponding photon is thus ${\vec{s}}_{0}={\vec{r}}_{f}-{\vec{r}}_{0}$. At this point, it should be pointed out that there are also a number of different strategies for the modelling of Gaussian beams within MC simulations. For example, using the Box–Muller transformation, the starting point on the lens can be sampled according to a Gaussian distribution and then started towards a random point within the beam waist, as in [21]. There are also methods where the photons are propagated along hyperbolic branches until the first interaction to mimic a Gaussian beam [22,23]. However, such an approach would be impractical for the ray-based intersection calculation used here. Since an initial Gaussian intensity profile and a focal spot of diffraction-limited size had no significant effect on the image characteristics of a single cylinder, they were omitted in the following for reasons of computational time and to limit the variables of influence.

After initialisation, the photons are propagated through the object using the well-known MC methodology. If a reflected photon hits the lens at a position $\left(x_{1},y_{1},z_{f}+2f\right)$, the location in the detection plane $\left(x_{2},y_{2},z_{f}+4f\right)$ is calculated using the ABCD matrix formalism. In particular, the refraction at a thin lens and the translation by 2f are taken into account:

(5) $$x_{2}=x_{1}+2f\tan\alpha_{x2}\;,$$

(6) $$y_{2}=y_{1}+2f\tan\alpha_{y2}\;,$$

where

(7) $$\tan\alpha_{x2}=-\frac{x_{1}}{f}+\tan\alpha_{x1}\;,$$

(8) $$\tan\alpha_{y2}=-\frac{y_{1}}{f}+\tan\alpha_{y1}.$$

$\alpha_{x1}$ and $\alpha_{y1}$ are the angles between the incident light ray and the optical axis in *x*-*z* and *y*-*z* projection, respectively. Depending on whether the ray has a positive or negative slope in the respective projection, $\alpha_{1}$ is considered positive or negative according to the sign convention of the ABCD matrix method. If a ray of light falls within the radius of the pinhole, i.e., $\sqrt{{x_{2}}^{2}+{y_{2}}^{2}}<r_{\mathrm{pin}}$, it will finally be detected. This numerical procedure will be repeated until the maximum number of photons *N* has been reached.

### 2.4. Maxwell-Based Calculation of the CLSM Images

To simulate a confocal microscope image of a single cylinder based on the solution of Maxwell’s equations, a similar approach was followed as described by Weise et al. [17] for spheres. It was assumed that the problem is approximately invariant along the cylinder axis (*x*-axis) to reduce the computational burden. This leads to a two-dimensional problem in the *y*-*z* plane. Furthermore, polarisations parallel and perpendicular to the plane of interest do not mix in a two-dimensional system, and for each polarisation, the image can be computed using a scalar approach. The confocal image amplitude U(y,z) can thus be calculated approximately as

(9) $$U\left(y,z\right)=\int_{-k_{y,\max}}^{k_{y,\max}}\int_{-k_{y,\max}}^{k_{y,\max}}S\left(k_{y},k_{y}^{\prime }\right)\exp\left\{i\left[z\left(k_{z}^{\prime }-k_{z}\right)+y\left(k_{y}^{\prime }-k_{y}\right)\right]\right\}dk_{y}dk_{y}^{\prime }\;,$$

where $S(k_{y},k_{y}^{\prime })$ is the scattering function of the imaged cylinder for perpendicular or parallel polarisation, obtained from the solution of Maxwell’s equations for the scattering by a cylinder [24]. $k_{z}$ and $k_{z}^{\prime }$ can be written as $k_{z}=\sqrt{k^{2}-k_{y}^{2}}$ and $k_{z}^{\prime }=\sqrt{k^{2}-{k_{y}^{\prime }}^{2}}$ with the absolute value of the wave vector $k=|\vec{k}|$. In Equation (9), it was also assumed that the exciting and the collecting light paths are equal and that the pupil functions are constant between the maximum *k* values allowed by the numerical aperture NA, otherwise zero. Therefore, the integration limits in Equation (9) are defined by $k_{y,\max}=k\;\cdot \;\mathrm{NA}$. In order to obtain a confocal image of a cylinder, Equation (9) was solved numerically for parallel and perpendicular polarisation by means of a Riemann sum. The average unpolarised intensity was calculated by

(10) $$I\left(y,z\right)=\frac{1}{2}\left({\left|U_{p}\left(y,z\right)\right|}^{2}+{\left|U_{s}\left(y,z\right)\right|}^{2}\right)\;,$$

where $U_{p}\left(y,z\right)$ and $U_{s}\left(y,z\right)$ are the image amplitudes for parallel and perpendicular polarisation, respectively.

## 3. Results

### 3.1. Theoretical Comparison

**Figure 3:**
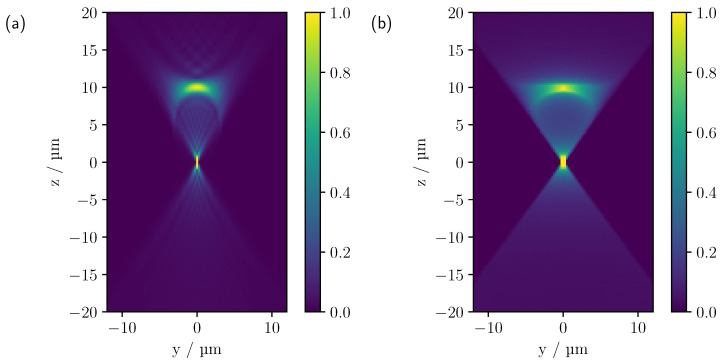
Theoretical comparison between CLSM images computed using (**a**) Maxwell’s theory and (**b**) MC considering two-dimensional light propagation. Both calculations were made for a cylinder with a radius of 10 μm and a refractive index of 1.6285, whose geometrical centre was at (y=0,z=0). The NA was set to 0.6. For the calculation of the confocal scan by means of Maxwell’s theory, a wavelength of 552 nm was assumed.

Prior to the simulation of more complex multi-cylinder phantoms by means of MC simulations, the extent to which the image characteristics of the Maxwell calculation can be reproduced should first be investigated in a qualitative manner. For this theoretical comparison, CLSM images were computed for a single cylinder using the MC and Maxwell methods, as described in Section 2.3 and Section 2.4. Since the theoretical calculation based on Maxwell’s equations only considers a two-dimensional illumination and detection geometry, the MC simulation was adapted for this case for better comparability. The cylinder was given a radius of 10 μm and a refractive index of n=1.6285. Moreover, the NA was set to 0.6 and a wavelength of 552 nm was selected. The size of the pinhole in the MC simulation was chosen to be one Airy unit (AU). The spatial discretisation by means of tetrahedra in the MC simulation was adjusted to such a fine degree that no deviations from an analytically implemented cylinder could be detected. As the Maxwell calculation showed that the cylinder radius had a significant effect on the image characteristics even with small changes due to interference effects, an average of 11 calculated images was made for cylinder radii in the interval (10.00±0.25) μm. Figure 3 shows the theoretical comparison, with both calculations normalised to the first intensity maximum at the surface z=10μm.

Both calculations show an hourglass-like intensity pattern, with a prominent intensity maximum at the surface at z=10 μm and a central maximum in the middle of the cylinder slice at z=0 μm. As the chosen scanning plane resembles a section through a sphere, it stands to reason that similar image features are observed, e.g., as in [15]. As in the case of the sphere [17,25], it can also be seen that the central maximum widens according to the point spread function (PSF). Geometric optics shows that the appearance of the peak intensity at z=10 μm is mainly due to reflections at the upper surface of the cylinder. More precisely, at (y=0 μm,z=10 μm), where the surface normal is exactly along the optical axis, all reflected rays, obeying the law of reflection, hit the lens again, symmetrically to the corresponding incident ray, and are finally detected. As the focus moves away from this point, fewer rays meet the detection condition and the measured intensity decreases. The maximum intensity in the centre (y=0 μm,z=0 μm) is caused by rays that intersect the surface, where the direction of propagation of the rays is along the surface normal. The rays are then either directly reflected and detected, or transmitted and, after being reflected opposite the point of entry, finally reach the detector if they have been transmitted again. Nevertheless, deviations can be seen, in particular in the central signal and at the edge of the hourglass-like intensity profile, which appears to have a smaller angle in the Maxwell calculation than in the MC simulation. This is probably due to interference effects causing a reduction in the lateral intensities of the hourglass-like pattern, giving it a narrower appearance. On the other hand, the greater extension of the central maximum within the MC-simulated CLSM scan probably reflects differences in detection methodology. In the MC calculation, the backscattered light is focused by a lens onto a pinhole, where the pinhole radius determines the acceptance range of the detected light. In contrast, the Maxwell computation takes no further imaging optics into account and only detects light corresponding to the *k* values allowed by the NA. Consequently, deviations are expected in the central focus-like spot that maps changes in the PSF.

### 3.2. Variation of the Refractive Index Difference

The experimental CLSM image stacks were acquired as described in Section 2.1 at a resolution of 1024×1024×800 pixels by scanning the focus of the incident beam in all 3 directions. The two multi-cylinder geometries were measured both with an air objective with NA=0.6 and with an oil objective in immersion oil with NA=1.4. Considering both materials used for 3D printing and assuming a refractive index of n=1.5180 for the immersion oil, the differences in refractive index Δn for the individual measurements are 0.6285, 0.5133, 0.1105 and −0.0048. As the image characteristics are similar to those at Δn=0.6285, the measurements at a refractive index difference of 0.5133 are not considered in the following. For better statistics, all measurements were averaged over 40 scans along the *x*-axis. In addition, the background noise was subtracted line by line. The resulting images were then normalised to the maximum intensity. Further filtering using a moving average with a window width of 5 pixels was applied to the measurements with the smallest refractive index difference of n=−0.0048 to improve the signal-to-noise ratio. In this case, due to strong reflections on the glass substrate, the intensity was normalised to the signal from the first cylinder surface.

For the simulation, *y*-*z* scans were computed with a resolution of 201×201 pixels for the smaller NA and 201×401 for the larger NA. In order to improve the visibility of the signals, an up-scaling of the lateral resolution by a factor of two has been applied by means of cubic interpolation. The number of photons per pixel for a refractive index difference of 0.6285 and 0.1105 was $N=10^{7}$, while for the lowest refractive index difference Δn=−0.0048, the photon count was $N=10^{8}$ to compensate for the low reflectance. The scattering coefficient $\mu_{s}$ and the absorption coefficient $\mu_{a}$ were fixed to zero for all simulations neglecting volume scattering and absorption. Using the surface meshes created for 3D printing, tetrahedral meshes were generated to model the multi-cylinder structures, excluding the support frames around the cylinders required for 3D printing. The spatial discretisation of the cylinders using tetrahedra was fine-tuned so that no deviations from analytically implemented cylinders could be detected. Based on the experimental setup, a numerical aperture NA=0.6, focal length f=2mm and pinhole radius $r_{\mathrm{pin}}=1.68\mu m$ were assumed for the simulation with air as the surrounding medium (Δn=0.6285). For the remaining simulations with immersion oil as the external medium (Δn=0.1105 and −0.0048), the parameters NA=1.4, f=0.3mm and $r_{\mathrm{pin}}=0.72\mu m$ were used.

**Figure 4:**
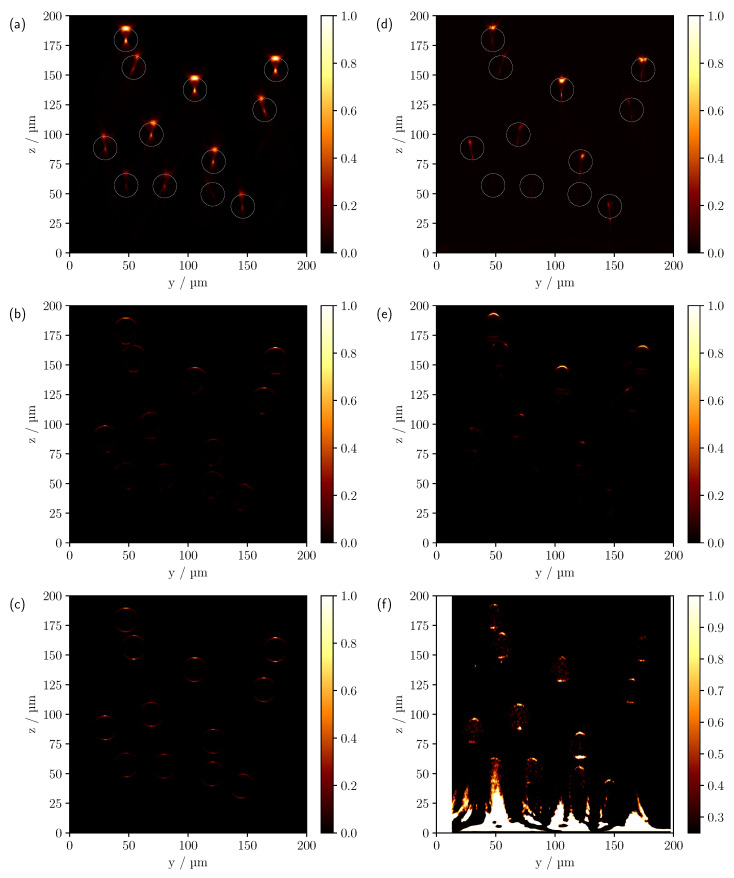
Comparison of the simulated (**a**–**c**) and measured (**d**–**f**) CLSM images of the multi-cylinder phantom with individual cylinder radii of 10μm. The difference in refractive index $\Delta n=n_{cyl}-n_{s}$ from top to bottom is 0.6285, 0.1105 and −0.0048. The white circles in (**a**,**d**) indicate the theoretical cylinder positions.

The comparison between simulated (left panel) and measured (right panel) CLSM scans can be seen in Figure 4 for different refractive index differences for the multi-cylinder geometry with cylinder radii of 10 μm.

**Figure 5:**
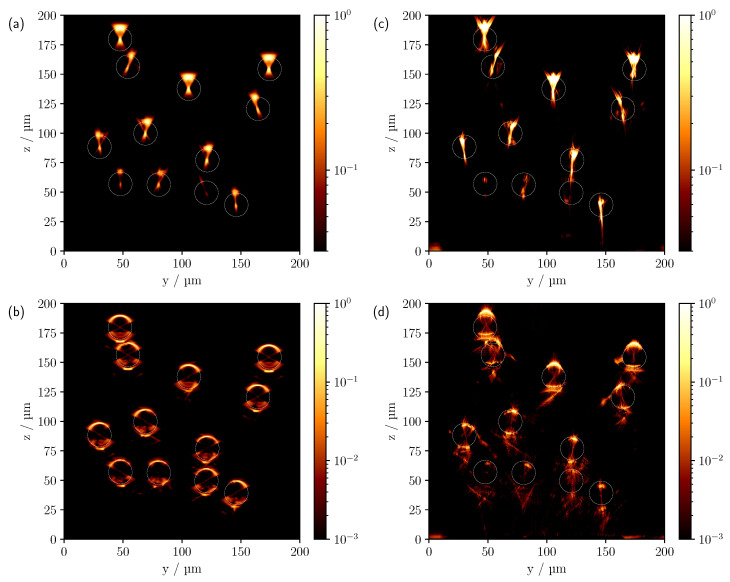
Comparison of the simulated (**a**,**b**) and measured (**c**,**d**) CLSM images of the multi-cylinder phantom with individual cylinder radii of 10μm in logarithmic scale. The difference in refractive index $\Delta n=n_{cyl}-n_{s}$ from top to bottom is 0.6285 and 0.1105. The white circles indicate the theoretical cylinder positions.

Starting with measurements in air and Δn=0.6285, first row, a good agreement between theory and experiment can be observed. In both Figure 4a,d, the first free-standing cylinders show a reflection on the surface and a central focusing point inside the cylinders, which was expected from the results of the comparison made in Figure 3. As a consequence of the significant difference in refractive index to the surrounding medium, the signals from the lower cylinders are shadowed by the cylinders above them. Three of the four lowest cylinders are barely visible in the experimental scan, presumably due to the presence of surface roughness of the superjacent cylinders caused by the printing process and the resulting higher scattering in the backward direction. In addition, despite the laterally enlarged support frame of the 3D print, there may be an effective reduction in NA due to shadowing, especially for the outer and deeper cylinders, and consequently an intensity decrease with depth. It can also be seen that the theoretical cylinder positions shown in white circles do not correspond exactly to the measured positions, which can be explained by a tilted sample. An examination of the *x*-*z* scans confirmed this assumption. Note that due to the alignment of the cylinders along the *x*-axis, the image features in the *x*-*z* projection are invariant along *x*, provided the cylinders are ideally aligned along the *x* direction. In order to make the image features more apparent, a logarithmic representation of the comparison is shown in Figure 5a,c. To compensate for the increased experimental intensity attenuation in the logarithmic plot, each pixel was multiplied by an empirically determined factor of five in Figure 5c, indicating a good qualitative agreement also for the lowest cylinders.

**Figure 6:**
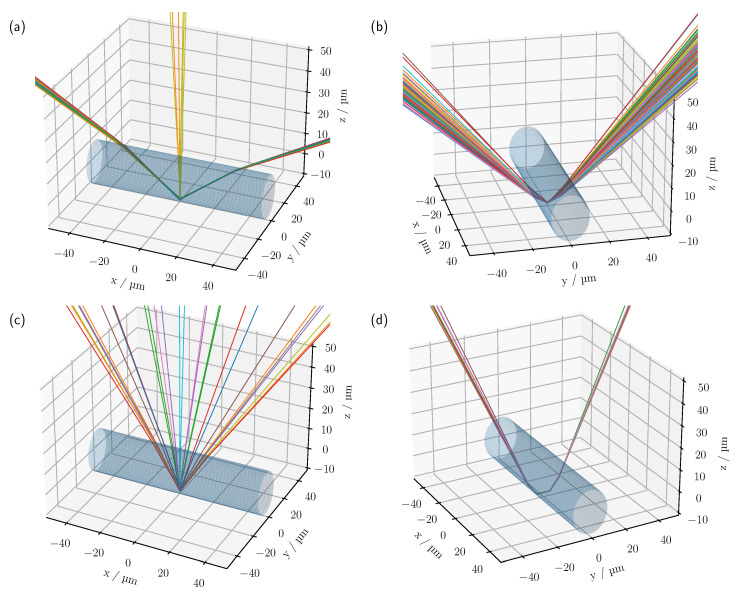
Example of detected photon paths from the MC simulation for a cylinder aligned along the *x*-axis with a radius of 10 μm and a CLSM focus at (**a**) (0,0,−4.5μm), (**b**) (0,0,−8μm), (**c**) (0,0,−12μm) and (**d**) (0,0,−19.5μm). The simulation parameters have been set to NA=1.4, Δn=0.1105 and $r_{\mathrm{pin}}=0.72\mu m$. Only a section of the full cylinder length of 200 μm is shown.

With the reduction of Δn to 0.1105, see Figure 4b,e, each cylinder in the model can be clearly identified in the simulated CLSM image. The higher NA of the oil objective results in a lateral broadening of the surface reflexes. However, compared to the experiment, the simulated surface reflection shows a maximum in the central area of the top of the cylinder, where the surface normals are still approximately parallel to the *z*-axis, with the intensity decreasing towards the outside. This is to be expected when geometric optics are considered. As there are no ideal surfaces in the experiment as is the case in the simulation, the surface reflection also shows a higher intensity laterally. In addition, a signal can now be seen on the underside of the cylinders in Figure 4b,e. Note that previously visible signals, such as the central focus-like spot as in Figure 4a,d, are not visible in this linear representation because the ratio of the different feature intensities varies significantly as the optical system parameters and the reflectance index difference change. As with the measurement in air, there is a faster decrease in intensity with depth in the experiment. Again, increased surface scattering may be a factor. With the higher NA of the oil objective, the surrounding support structures of the phantom are also expected to reduce the effective NA and therefore the detected intensity compared to the simulation, especially for lower-lying cylinders. This is particularly clear for cylinders at the edge that are only partially shadowed by cylinders above them, such as the cylinder at (y=30 μm,z=88 μm) in Figure 4b. Yet, in comparison to the upper row of cylinders, only a very faint signal can be detected. Without shadowing from the support structures, a clearly discernible signal from the outer surface would be expected in such a case. Other effects that cannot be described by the ideal numerically implemented optical system, such as various aberrations and a Gaussian beam, could also contribute to this signal reduction. After compensating for the increased intensity drop by pixel-wise multiplication by an empirically determined factor of three and the logarithmic display, see Figure 5d, most of the lower-lying cylinders are now visible in the experimental scan. The logarithmic plot reveals further reflexes on the underside of the cylinders. One prominent signal appears about 8 μm below the centre of the cylinder, as already seen in the linear view, and a second about 2 μm below the lower surface of the cylinder; see, for example, the cylinder located at (y=106 μm,z=138 μm). Analysing the individual photon paths within the MC simulation shows that these two signals mainly occur as follows: refraction on the surface of the cylinder, reflection on the underside of the cylinder and, after further refraction, incidence on the lens, see Figure 6b,c.

**Figure 7:**
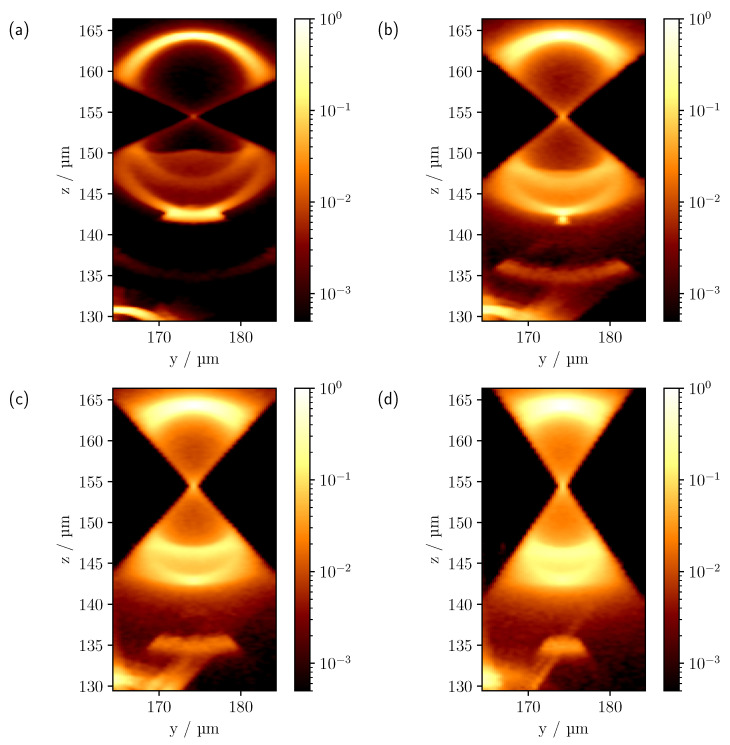
Variation of the NA for a section of Figure 5b: (**a**) NA=1.4, (**b**) 1.2, (**c**) 1.0 and (**d**) 0.9.

For the reflex that results from a CLSM focus at about (y=0,z=−8 μm), see Figure 6b, the rays enter and leave the cylinder mainly laterally. In contrast, for a light source focused at (y=0,z=−12 μm), the rays tend to be directed along the cylinder axis, see Figure 6c. Apart from these 2 features, another discrete signal is visible for the simulation in Figure 5b at about 4.5 μm below the centre of the cylinder. Again, the trajectories of the rays tend to follow the length of the cylinder, but at a larger angle than in the previous case, see Figure 6a. In addition, the measurements in Figure 5d show a focus-like point below the cylinders, which is not visible in the simulations; for example, see the cylinder located at (y=54 μm,z=156 μm). The reason for this could again be a reduced effective NA in the experiment. An important indication of this is the decreased extent of the hourglass-shaped intensity profile in the experimental scan compared to the calculated image. For example, when the NA is reduced within the simulation, a similar focus-like spot could be reproduced below the cylinders in addition to a narrowing surface signal, see Figure 7.

Shading caused by the supporting structure would effectively reduce the NA and explain the observed variations. The real Gaussian intensity distribution and deviations from the ideal optical system could also lead to a reduction in the effective NA compared to the simulation. Given that a larger NA also includes the angular range of a smaller NA, and provided that there are no interference effects responsible for the formation of this signal, it is expected that rays will reach the detector in the simulated scan when the CLSM is focused 19.5 μm below the centre of the cylinder, as seen in Figure 6d and Figure 7a. However, since at NA=1.4 the intensity of the signal located about 19.5 μm below the cylinder centre is orders of magnitude below the intensity of the remaining signals, it is not visible in the scaling chosen in Figure 5b. Therefore, if geometrical optics is consulted once again, the formation of the lower focus-like signal can be traced back to multiple reflections inside the cylinder, see Figure 6d. In general, it should be noted that the intensity characteristics of a single cylinder are very sensitive to the optical properties and parameters of the imaging system, as can be seen in Figure 7.

For the smallest refractive index difference of Δn=−0.0048, see Figure 4c,f, all cylinders are now clearly visible in both the simulated and measured CLSM image, even in the linear representation. Note, however, that intensity values less than 0.25 were truncated in the experimental scan for better comparability of the image features. The reason for this will be discussed below. Both scans show intensities only at the upper and lower interfaces to the medium owing to the minimal refraction. Since the difference in refractive index between the surrounding medium and the glass substrate located at z=0 μm is about an order of magnitude greater than the difference in refractive index between the medium and the cylinders, pronounced artefacts due to reflections from the glass surface can be seen in the lower part of the image in case of the experimental results, see Figure 4f. Overall, an increase in penetration depth was observed as the refractive index difference decreases. Qualitatively, this was explained in the previous discussion of the results by the reduction in reflectivity and lower refraction, with the implication that graphically speaking, the irradiated focus becomes less dispersed as it passes through the phantom and is, therefore, more preserved as it reaches the deeper-lying cylinders. Quantitatively, this observation can also be understood in terms of the scattering by a cylinder based on solving Maxwell’s Equations [24], more specifically by calculating the scattering efficiency and scattering functions for the different cylinder radii and refractive index differences being investigated. When looking at the scattering efficiency first, it is about 3 times higher for the largest refractive index difference of 0.6285 than for the smallest refractive index difference of −0.0048. However, the effect on the scattering function is far more critical; more specifically, when the refractive index difference is reduced from 0.6285 to −0.0048, the ratio between forward to backward scattering increases dramatically. There is an intensity decrease of about 2 orders of magnitude between forward and backward scattering for Δn=0.6285, 4 orders of magnitude for Δn=0.1105 and 7 orders of magnitude for Δn=−0.0048. In essence, by reducing the refractive index difference, proportionally less light is scattered backwards, allowing more light to reach the lower-lying cylinders.

**Figure 8:**
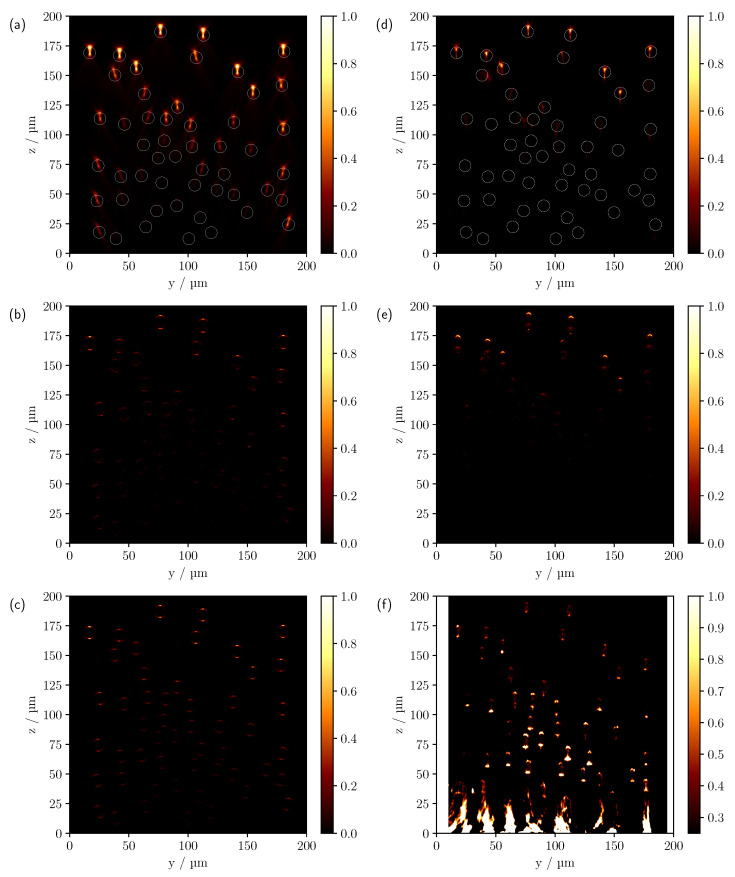
Comparison of the simulated (**a**–**c**) and measured (**d**–**f**) CLSM images of the multi-cylinder phantom with individual cylinder radii of 5 μm. The difference in refractive index $\Delta n=n_{cyl}-n_{s}$ from top to bottom is 0.6285, 0.1105 and −0.0048. The white circles in (**a**,**d**) indicate the theoretical cylinder positions.

If the phantoms with individual cylinder radii of 5 μm are now considered, which approximately halves the one-dimensional scattering cross section compared to the larger cylinders, the reduced cylinder size allows the number of individual scatterers to be increased from 12 to 51 within the volume of interest, see Figure 8. As a result, there are now about four times as many scatterers and a change in the scattering function can also be anticipated.

**Figure 9:**
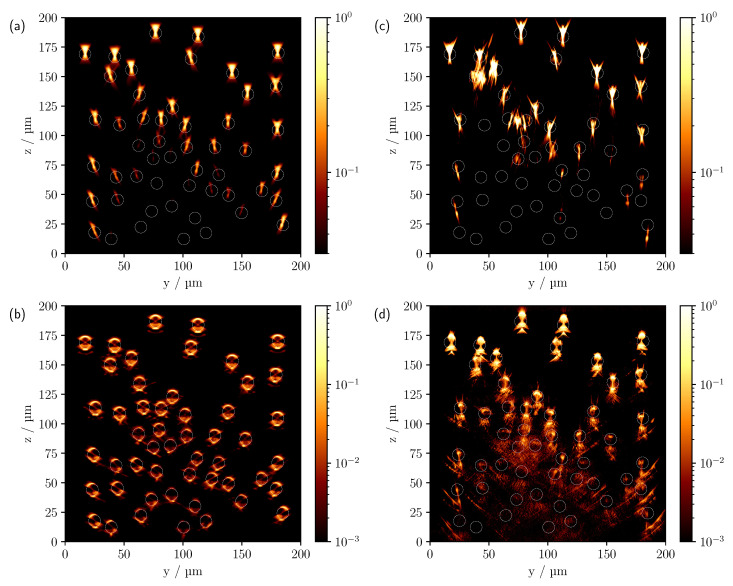
Comparison of the simulated (**a**,**b**) and measured (**c**,**d**) CLSM images of the multi-cylinder phantom with individual cylinder radii of 5 μm in logarithmic scale. The difference in refractive index $\Delta n=n_{cyl}-n_{s}$ from top to bottom is 0.6285 and 0.1105. The white circles indicate the theoretical cylinder positions.

At the largest refractive index difference of 0.6285, see Figure 8a,d, a reduction in penetration depth compared to the previous phantom can be observed in both the simulation and the measured scan as a result of the increase in scatterers. In addition, for the smaller cylinder radii, there is also a subtle blurring of the individual intensity features, as the pinhole size is now larger in proportion to the dimension of the cylinder. Furthermore, for the phantom with individual cylinder radii of 5μm the experiment shows that the intensity decreases more strongly with increasing depth, except for the measurements with the smallest refractive index difference. As previously discussed, this is likely to be due to a number of factors, including additional surface scattering, shading caused by the support geometry and deviations from the ideal assumed optical system. The shading appears to be one of the main causes when looking at the side cylinders in Figure 8a,d. The outer cylinders are only partially shadowed by the other cylinders above them. Therefore, partial intensity features are to be expected beyond the shaded regions. Comparison with the simulation, which shows clearly visible signals for the lateral cylinders, confirms this. Nevertheless, the logarithmic plot, Figure 9a,c, shows that after compensating for the increased intensity loss by multiplying the experimental scan by an empirically determined constant factor of five, the image features now show good agreement with the simulation.

**Figure 10:**
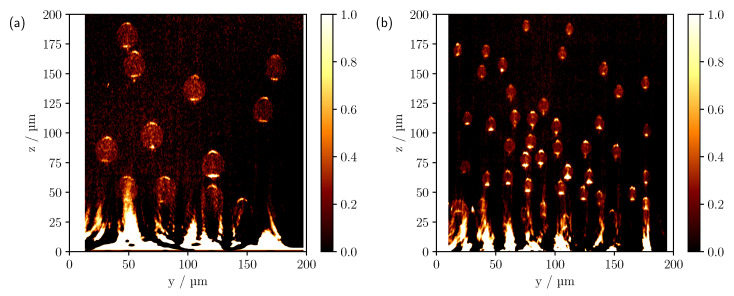
Comparison of measured CLSM images for cylinder radii of (**a**) 10μm and (**b**) 5μm with a difference in refractive index of −0.0048. Volume scattering becomes apparent when considering the full range of values.

Looking at the refractive index difference of 0.1105, Figure 8b,e, one can also see the deviations already discussed, especially the barely visible cylinders at the edge of the phantom. As far as the signal characteristics are concerned, once again a good agreement between the simulation and the experiment can be observed in the linear representation. Again, only the lower focus-like point is missing in the simulation. As with the cylinder phantom with radii of 10 μm at a refractive index difference of 0.1105, there are obvious differences between the theory and the measurement when the CLSM scans are plotted on a logarithmic scale, see Figure 9b,d. Here, too, an effective reduction in the NA would provide an explanation for the differences. For the smallest refractive index difference, see Figure 8c,f, apart from the artefacts caused by reflections from the glass surface, all cylinders are again visible in both scans despite the increase in scatterers. However, in the upper right corner, see Figure 8f, a cylinder appears to have been broken off during the printing or measuring process. As for the larger cylinders, values smaller than 0.25 were not taken into account for the presentation of the measurements with Δn=−0.0048, which was motivated by the following. When the full range of values is considered, the contribution of volume scattering within the cylinders becomes visible, see Figure 10. This has been neglected in the comparison between the experiment and simulation as no scattering in the cylinder volumes was assumed in the latter. Due to the short path lengths and the completely filled cylinder volumes in 3D printing, no significant contribution from volume scattering is expected in principle and is negligible compared to the surface contributions, especially for measurements with higher refractive index differences. Nevertheless, for the smallest refractive index difference, the reflectance caused by the cylinder surface decreases significantly and the volume scattering signal component becomes comparatively more relevant to the overall image. The cause of the volume scattering is likely to be minimal heterogeneities in the cylinder volume created during the printing process. As seen previously for the larger cylinders, the ratio of forward to backward scattering also increases significantly for the smaller cylinders as the refractive index difference decreases to Δn=−0.0048. Although in this case, the scattering efficiency increases about tenfold from a refractive index difference of −0.0048 to 0.6285, the influence of the refractive index difference on the phase function is still more substantial and would also explain the increase in penetration depth here, which is analogous to the larger cylinders.

### 3.3. Variation of the Numerical Aperture and Pinhole Size

To experimentally investigate the influence of numerical aperture and pinhole size on the individual characteristics of the CLSM scans, the measurement and evaluation routines already described in Section 2.1 and Section 3.2 were used. All scans were performed on the phantom with single cylinder radii of 10 μm with air as the surrounding medium.

**Figure 11:**
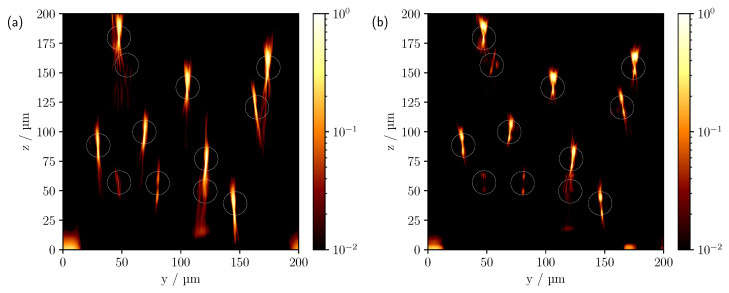
Comparison of measured CLSM images for a NA of (**a**) 0.3 and (**b**) 0.4 with a pinhole size of 1.0 AU.

Figure 11 shows the comparison between measurements at NA=0.3 and 0.4 on a logarithmic scale.

**Figure 12:**
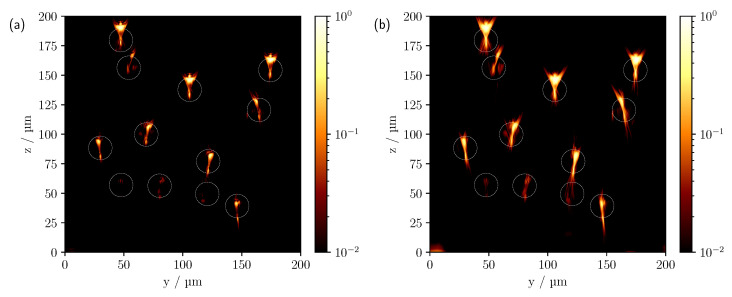
Comparison of measured CLSM images for a pinhole size of (**a**) 0.5 AU and (**b**) 2.0 AU with a NA of 0.6.

As shown in the previous section, a widening of the hourglass-like intensity distribution of a single cylinder can be observed as NA increases, with the angle approximately matching the incident geometric aperture. Correspondingly, a minimal spread is also expected here, as can be seen in Figure 11, especially for the upper row of cylinders. A further enlargement of the NA and thus a broadening of the intensity profile is shown in Figure 5c. It is also noticeable that in Figure 11 at NA=0.3 there is a blurring and elongation of the reflections in comparison to the larger NA. This is due to the different sizes of the pinholes that result from the different NA. The image-side pinhole radius was about 33.7 μm for the 10× objective (NA=0.3) and about 50.5 μm for the 20× objective (NA=0.4). Considering the magnification, the pinhole radius on the object side was therefore about 33% larger with the smaller NA than with an NA of 0.4. Furthermore, when measuring with a larger NA, additional reflexes may be seen due to the wider angle of illumination. For example, for the cylinder located at about (y=54 μm,z=156 μm), see Figure 11b, a partial signal can still be observed, whereas this is no longer the case for measurements at the smaller NA, see Figure 11a. Geometrically, this is straightforward, since the angular range of the incident light that contributes to the formation of this signal is no longer included in the latter. In Figure 12, the pinhole size was explicitly varied while keeping the NA constant.

As expected, the enlargement of the pinhole leads to a blurring of the image features. The expansion of the intensity signals is also increased. This results in better visibility, especially for the lower cylinders.

## 4. Conclusions

Image formation in a CLSM was investigated for 3D printed multi-cylinder phantoms with cylinder radii of 5 μm and 10 μm and for different refractive index differences to the surrounding medium, both experimentally and theoretically. As a preliminary validation, the MC implementation of CLSM was compared with a Maxwell calculation in two dimensions for a single cylinder and good qualitative agreement was found. For the largest refractive index difference, i.e., air as the external medium, measurements and simulations showed high agreement in the image features. When the refractive index difference was reduced to 0.1105, there were noticeable deviations in the comparison between theory and experiment, presumably due to the support structure of the real cylinder models and the resulting effectively reduced NA. For the smallest refractive index difference of −0.0048, the signal characteristics of the individual cylinders again showed good agreement. The increase in penetration depth could also be reproduced. In the experiments with the lowest refractive index difference, a volume scattering contribution was observed in addition to the signal contributions from the cylinder surfaces. Finally, the influence of the NA and pinhole size on the image features was investigated experimentally. One possible application of the phantoms presented in this paper could be in the verification and optimisation of CLSM devices. In further investigations, the agreement between theory and experiment could be improved, in particular by more precise modelling of the real optical system, e.g., including a Gaussian beam model and ray tracing through the real measurement setup, but also by a complete consideration of the real samples including possible support structures, microscope slides and surface roughness of the cylinders. The latter could be characterised, for example, by goniometric measurements and subsequent comparison with solutions of Maxwell’s equations in order to implement a corresponding model in the MC simulation. Moreover, the multi-cylinder phantoms could be measured using optical coherence tomography and compared with simulations, e.g., based on Maxwell’s equations [26,27]. Additional experimental investigation of the samples using other 3D imaging techniques such as white light interferometry or optical sectioning structured illumination microscopy [28,29,30] would also be conceivable.

## Data Availability

Data sharing is not applicable to this article.
